# Reference Equation for Respiratory Pressures in Pediatric Population: A Multicenter Study

**DOI:** 10.1371/journal.pone.0135662

**Published:** 2015-08-20

**Authors:** Fernanda Cordoba Lanza, Mara Lisiane de Moraes Santos, Jessyca Pachi Rodrigues Selman, Jaksoel Cunha Silva, Natalia Marcolin, Jeniffer Santos, Cilmery M. G. Oliveira, Pedro Dal Lago, Simone Dal Corso

**Affiliations:** 1 Postgraduate Program in Rehabilitation Sciences, Universidade Nove de Julho - UNINOVE, Sao Paulo, SP, Brazil; 2 School of Physical Therapy, Universidade Federal do Mato Grosso do Sul, Campo Grande, MS, Brazil; 3 School of Physiotherapy, Health Department, Universidade Nove de Julho - UNINOVE, Sao Paulo, SP, Brazil; 4 Universidade Federal de Ciências da Saúde de Porto Alegre (UFCSPA), Laboratory of Physiology, Porto Alegre, RS, Brazil; 5 State University of Healthy Science of Alagoas, (UNCISAL), Maceió, AL, Brazil; University Children's Hospital Basel, SWITZERLAND

## Abstract

Previous studies have proposed only one prediction equation for respiratory muscle strength without taking into consideration differences between ages in pediatric population. In addition, those researches were single-center studies. The objective of this study was to establish reference equations for maximal inspiratory pressure (PImax) and maximal expiratory pressure (PEmax) in children and teenagers. In a multicenter study, 450 healthy volunteers were evaluated (aged 6–18yrs). There were included volunteers with normal lung function. We excluded volunteers who could not perform the tests; participated in physical activity more than twice a week; were born prematurely; smokers; chronic respiratory, cardiologic, and/or neurologic diseases; had acute respiratory disease during the prior three weeks. The volunteers were divided into two groups: Group 6–11 (6–11yrs) and Group 12–18 (12–18yrs). PImax and PEmax were measured according to statement. The mean PImax value was 85.6 (95%IC 83.6–87.6 cmH_2_O), and PEmax 84.6 (95%IC 85.5–86.2 cmH_2_O). The prediction equations for PImax and PEmax for Group 6–11 were 37.458–0.559 + (age * 3.253) + (BMI * 0.843) + (age * gender * 0.985); and 38.556 + 15.892 + (age * 3.023) + (BMI * 0.579) + (age * gender * 0.881), respectively (R^2^ = 0.34 and 0.31, P<0.001). The equations for Group 12–18 were 92.472 + (gender * 9.894) + 7.103, (R^2^ = 0.27, P = 0.006) for PImax; and 68.113 + (gender * 17.022) + 6.46 + (BMI * 0.927), (R^2^ = 0.34, P<0.0001) for PEmax. This multicenter study determined the respiratory muscle strength prediction equations for children and teenagers.

## Introduction

The measurement of maximal inspiratory pressure (PImax) and maximal expiratory pressure (PEmax) is a simple and noninvasive technique by which to evaluate respiratory muscle strength [1]. This method can provide important information in clinical practice regarding patients with pulmonary and extra-pulmonary diseases [2–8]. Some lung diseases [4–6,8], such as asthma and cystic fibrosis, are comorbid with reduced respiratory muscle strength caused by lung function deterioration. The influence of respiratory co-morbitities can produce similar conditions in patients with hematology [2] and osteomusuclar [3,7] diseases. Beside this, measuring respiratory muscles strength helps to determine the benefits of respiratory muscle training[6–8]. PImax can also be studied as a predictive evaluation for successfully weaning children from mechanical ventilation systems [9].

Considering the importance of assessing respiratory muscle strength, several prediction equations for PImax and PEmax have been developed for children and teenagers in different countries [10–17]. One of the first of these equations was performed in Canada, in which Gaultier et al. [10] determined increases in respiratory muscle strength by age. In 2000, Wilson et al. [12] studied 135 British volunteers under 18 years of age and concluded that PImax and PEmax increases are related to age. Similar results were observed by Smyth et al., [13] but the authors suggested a need for measurement standardization. In 2003, Doménech-Clar et al. [14] and Matecki et al. [15] developed predicted equations for the Spanish and French pediatric populations, respectively. Those authors stated that reference values of respiratory muscle strength can be influenced by ethnicity, and thus, that regional values are preferable when determining normal values for PImax and PEmax.

To our knowledge, there have been two previous reports on PImax and PEmax reference equations for the Brazilian pediatric population. [16,17] However, these studies, as well as those described above, were carried out at a single center. Therefore, the aim of this study was to establish reference equations for PImax and PEmax in healthy children and teenagers by conducting a multicenter study.

## Materials and Methods

### Population

This research was a cross sectional and multicenter study conducted in four centers, one each in the southeast area, south area, center—west area, and northeast area of Brazil. Healthy subjects (normal lung function tests above 80% of predict, no respiratory or cardiovascular diseases) aged 6–18 years were recruited from private and public schools. We excluded volunteers who could not perform the tests or who had chest wall deformities; chronic respiratory, cardiologic, and/or neurologic diseases; participated in physical activity more than twice a week; were born prematurely; smokers; or had acute respiratory disease during the prior three weeks. A completed questionnaire on respiratory diseases and written informed consent were obtained from the legal guardians of all volunteers, and the local ethics committee, Research Ethics Committee of Universidade Nove de Julho, approved the study (number: 483692). This study was conducted in accordance with the amended Declaration of Helsinki.

The protocol started in August 2012 and finished in February 2014. All measurements were obtained at the volunteers’ schools or at the centers’ physiology laboratories by trained investigators. The total population was divided into two groups: Group 6–11 (volunteers 6–11 years of age) and Group 12–18 (volunteers 12–18 years of age).

### Measurements

Body mass index (BMI) was measured to the nearest 0.1 kg using a calibrated balance (110F; Welmy, São Paulo, Brazil), and body height (cm) was determined to the nearest 0.5 cm using a stadiometer. BMI was calculated as weight/height^2^.

Spirometry was performed with a calibrated pneumotachograph (CPFS/D USB; Medical Graphics, St. Paul, MN). The technical procedures and the acceptability and reproducibility criteria were as recommended by the American Thoracic Society/European Respiratory Society [18]. We recorded forced vital capacity (FVC), forced expiratory volume at the first second (FEV_1_), and FEV_1_/FVC. The measurements were compared with those predicted for the Brazilian population [19].

PImax and PEmax were obtained following guidelines [1]. Respiratory muscle strength was measured using an aneroid-type manometer (± 150cmH_2_O and ± 300 cmH_2_O; GeRar, São Paulo, Brazil). Before each measurement, the investigator explained the required maneuver and demonstrated it visually to the volunteer. The volunteers were standing, wearing nose clips, and with a rigid, plastic, flanged mouthpiece in place. A small leak was introduced between the occlusion and the mouth in order to prevent glottic closure, and the subjects held their hands to their cheeks during the maneuver. PEmax was measured after maximal inspiration (from total lung capacity), and PImax was measured after maximal expiration (from residual volume). The minimal duration of the maneuver was three seconds, and a plateau of at least two second was required. A resting period of one minute was allowed between each PEmax and PImax maneuver. The measurements were stopped when five maneuvers were concluded and at least three of them did not differ by more than 10%[1]. The best PImax and PEmax values were used for analysis.

The PImax and PEmax were used as outcomes to determine the sample power. The post hoc sample power was calculated using the G*Power 3.1 [20] program. The calculation required the R^2^ value observed in the study (0.27 to 0.34), the sample size (n = 318 for Group 6–11 and n = 132 for Group 12–18), the number of independent variables included in the model, and α value (0.05). After considering these variables, the sample power for both outcomes was 99%.

### Statistical analysis

The normality of data was analyzed by the Shapiro-Wilk test. The data showed parametric distribution and were expressed as mean and 95% confidence interval (95%CI). Unpaired t-tests were used to compare PImax and PEmax between gender; and PImax and PEmax between groups (Group 6–11 and Group 12–18). A multilinear regression analysis (stepwise) was performed for PImax and PEmax. For this analysis gender, age, weight, and height were considered as independent variables. The interactions between independent variables was tested in this model, the conditional (interaction) between age and gender, height and weight was tested. To adjust the between center difference, we included Dummy variables in the final model and these variables added constant values for the equation. Dummy coding is a way of representing groups of people using zero and one, considering the differences between groups. To do this, we created three Dummy variables, because we have four centers (k-1; Dummy variable is one less the number of the groups). The Southeast center was the reference group at Dummy variables. The coefficients and 95% confidence intervals are shown for each model. The probability of a type I error was established at 0.05 for all tests. The SPSS statistical package, version 22 (Chicago, IL) was used.

## Results

We evaluated 473 volunteers, of whom 23 were excluded (14 due to difficulties performing the respiratory muscle strength technique and nine due to lung function abnormality). The final sample consisted of 450 volunteers, 238 (53%) of whom were female. By center, 195 volunteers were from Southeast, 95 from South, 74 from Center West and 86 from Northeast. The volunteers from Center West and from Northeast did not perform the lung function test (160 [35%]). There was no difference on respiratory muscle strength between the volunteers who performed the lung function test and those who did not. The volunteers’ characteristics are described in Table 1.

**Table 1:** Characteristics of all the subjects (mean [95%CI]).

Variables	Total Group (n = 450)	Female (n = 238)	Male (n = 212)
Age (years)	10.4 [9.3–10.4]	10.3 [9.9–10.6]	10.0 [9.6–10.4]
Weight (Kg)	39.3 [37.9–40.7]	40.1 [38.2–42.0]	38.4 [36.3–40.3]
Height (cm)	142.2 [140.8–143.7]	142.7 [141.0–144.5]	141.6 [139.3–144.0]
BMI (Kg/cm^2^)	18.7 [18.3–19.1]	19.0 [18.5–19.6]	18.3 [17.8–18.8]
FVC, L^&^	2.6[2.5–2.7]	2.5[2.4–2.6]	2.7[2.5–2.8]
FVC (%pred)^&^	104.1[102.6–105.5]	103.5[101.5–105.3]	104.9[102.6–107.1]
FEV_1_, L^&^	2.3 [2.2–2.4	2.3[2.2–2.3]	2.4 [2.2–2.5]
FEV_1_ (%pred) ^&^	105.7 [105.7–107.2]	105.4[103.4–107.5]	106.0 [103.8–108.2]
FEV_1_/FVC^&^	91.0 [90.3–91.8]	91.3 [90.4–92.1]	90.7 [89.4–92.0]
PImax (cmH_2_O)	85.6 [83.6–87.6]	82.1 [79.2–84.5]*	89.7 [86.8–92.8]
PEmax (cmH_2_O)	84.6 [85.5–86.2]	80.2 [77.7–82.5]*	89.5 [86.4–91.9]

BMI: body mass index; FVC: forced vital capacity; FEV_1_: forced expiratory volume at the 1^st^ second; FEV_1_/FVC: relationship between FEV_1_/FVC; PImax: maximal inspiratory pressure; PEmax: maximal expiratory pressure.

^&^ of the 450 volunteers, 290 made spirometry, (159 [55%] female);

* *P* < 0,0001 *vs* Male.

**Fig 1:**
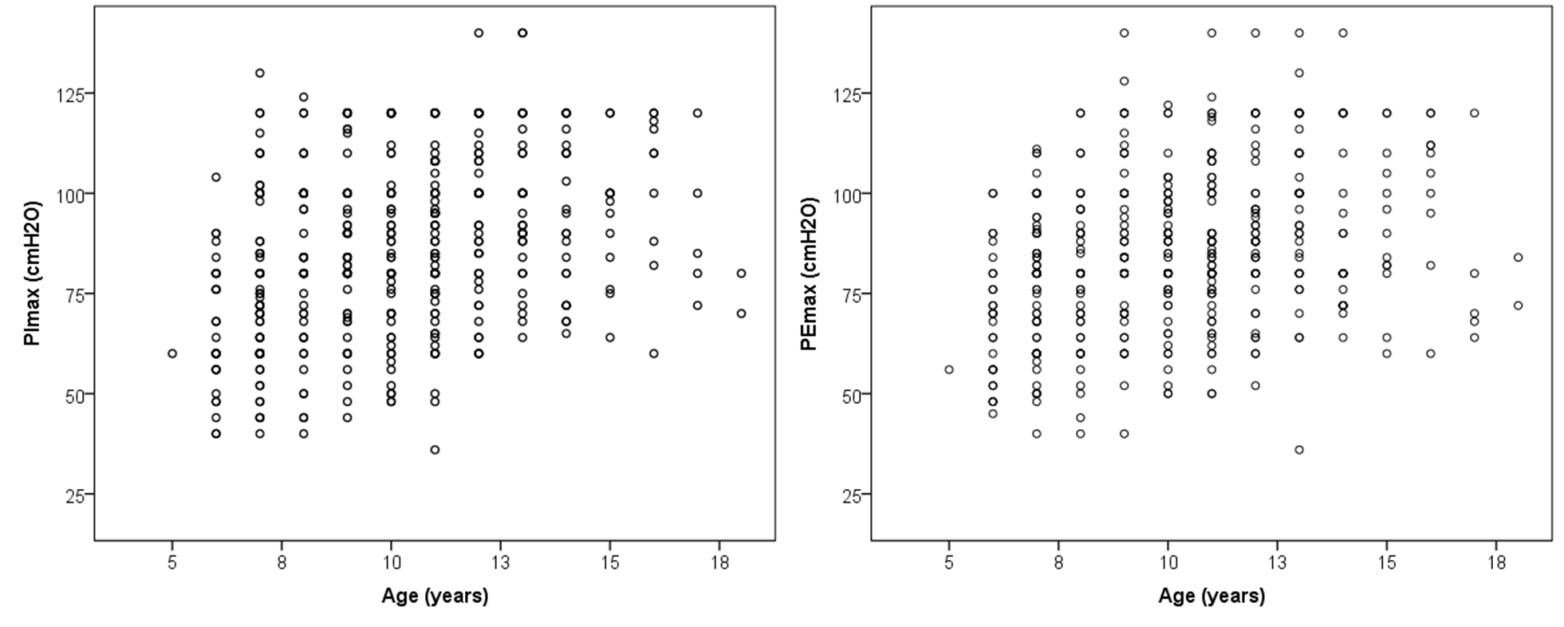
Scatter plot of respiratory muscles strength (PImax and PEmax) and age.

There was a statistically significant difference in respiratory muscle strength between male and female subjects (Table 1); PImax was 82.1 [79.2–84.5] cmH_2_O for females and 89.7 [86.8–92.8] cmH_2_O for males (P<0.0001) and PEmax was 80.2 [77.7–82.5] cmH_2_O for females and 89.5 [86.4–91.9] cmH_2_O for males (P<0.0001). The older volunteers (Group 12–18) showed greater respiratory muscle strength than Group 6–11 (P < 0.0001; Table 2), which means that respiratory muscle strength increases as children grow, but there is nonlinear increases on it (Figure 1).

**Table 2:** Characteristics of both groups (mean [95%CI]).

	Group 6–11	Group 12–18
	n = 318	n = 132
Age (years)	8.7 [8.5–8.9]	14.0 [13.2–14.5]*
Weight (Kg)	32.8 [31.6–33.9]	54.2 [51.7–56.7]*
Height (cm)	134.9 [133.6–136.2]	158.8 [157.1–160.5]*
BMI (Kg/cm^2^)	17.6 [17.2–18.0]	21.2 [20.5–22]*
PImax (cmH_2_O)	81.6 [79.3–84.0]	95.2 [91.8–98.5]*
PEmax (cmH_2_O)	81.6 [79.4–83.7]	91.3 [87.9–94.8]*

BMI: body mass index; PImax: maximal inspiratory pressure; PEmax: maximal expiratory pressure.

*P< 0.0001 *vs* Group 6–11.

We observed difference of respiratory muscle strength between center, but we added Dummy variables in the final model to adjust the between center difference. We performed two multilinear analyses according to the groups. The variables that persisted in the equation for respiratory muscle strength (PImax and PEmax) in Group 6–11 were age, gender, and BMI. The variables that persisted in the equation for Group 12–18 were gender to PImax; gender and BMI to PEmax. The coefficients and 95% confidence intervals of the models are showed in Table 3.

**Table 3:** Predictor variables for respiratory muscles (PImax and PEmax) obtained from multiple linear regression analysis.

	**Group 6–11**						
**PImax**	**β**	**95% CI**		**PEmax**	**β**	**95% CI**	
		**Lower limit**	**Upper limit**			**Lower limit**	**Upper limit**
Constant	37.458	23.904	51.012	Constant	38.556	25.283	51.830
Dummy 1	-11.707	-17.313	-6.102	Dummy 1	-0.399	-5.810	5.012
Dummy 2	14.770	8.212	21.328	Dummy 2	12.876	6.529	19.224
Dummy 3	-3.622	-10.436	3.193	Dummy 3	3.415	-3.155	9.985
Age	3.253	1.950	4.555	Age	3.023	1.763	4.283
BMI	0.843	0.228	1.457	BMI	0.579	-0.021	1.178
Interaction (age*gender)	0.985	0.542	1.429	Interaction (age*gender)	0.881	0.448	1.313
	**Group 12–18**						
**PImax**	**β**	**95% CI**		**PEmax**	**β**	**95% CI**	
		**Lower limit**	**Upper limit**			**Lower limit**	**Upper limit**
Constant	92,472	84.770	100.174	Constant	68.113	51.077	85.150
Gender	9.894	3.361	16.428	Gender	17.022	10.513	23.532
Dummy 1	-4.391	-12.460	3.677	Dummy 1	-6.426	-14.496	1.644
Dummy 2	7.634	-29.763	45.030	Dummy 2	16.426	-20.638	53.169
Dummy 3	3.860	-5.827	13.546	Dummy 3	-3.379	-13.572	6.814
				BMI	0.927	0.157	1.697

Gender = 0 for female, and 1 for male; BMI: body mass index = weight /height^2^

PImax: maximal inspiratory pressure; PEmax: maximal expiratory pressure.

Dummy 1: Southeast, Dummy 2: Center West, Dummy 3: South.

*Equations:*

**Group 6–11**

PImax:37.458 – 0.559 + (age * 3.253) + (BMI * 0.843) + (age * gender * 0.985)

$$R^{2}:\;0.34,\;p\;<\;0.001$$

PEmax: 38.556+ 15.892 + (age * 3.023) + (BMI * 0.579) + (age * gender * 0.881)

$$R^{2}:\;0.31,\;p\;<\;0.001$$

**Group 12–18**

PImax:92.472 + (gender * 9.894) + 7.103

$$R^{2}:\;0.27,\;p\;=\;0.006$$

PEmax:68.113 + (gender * 17.022) + 6.46 + (BMI * 0.927)

$$R^{2}:\;0.34,\;p\;<\;0.001$$

## Discussion

We evaluated the respiratory muscle strength of 450 children and teenagers 6 to 18 years of age in four different centers in Brazil. The female volunteers had lower PImax and PEmax values compared to the males, and the younger volunteers (Group 6–11) had lower respiratory muscle strength values compared to the older volunteers (Group 12–18). The independent variables that persisted in the respiratory muscle strength equations were age, gender and BMI for the youngest, and age and/or BMI for the oldest; these variables explain approximately 30% of the variances in PImax and PEmax.

The difference in respiratory muscle strength between genders has been reported by many authors [13,14,21–23]. As expected, PImax end PEmax were lower in girls than in boys of the present study. The most plausible explanation is that the hormonal differences between the genders produce greater amounts of muscle mass in males than in females [24].

The volunteers in Group 6–11 had lower PImax and PEmax values compared to Group 12–18, data that agreed with previous studies, but there is a nonlinear relationship between age and respiratory muscles, as observed in the Fig 1. Matecki et al. [15], in a longitudinal study of Caucasian volunteers, suggested that PImax continues to increase up to 17 years of age and PEmax increases up to 15 years. The authors justified these results based on the increase in muscle size with body growth, the maturation of neural influence, and the endocrine changes at different ages. Heinzmann-Filho et al. [16] also described the influence of age on respiratory muscle strength in a Brazilian pediatric population, the youngest volunteers had lower respiratory muscle strength compared to the oldest.

Arnall et al. [22] evaluated volunteers 6–14 years old and described one equation for PImax and PEmax for this wide age range. A similar age range was evaluated by Domenéch-Clar et al. [14] and Wilson et al., [12] who analyzed volunteers between 8–17 years and 7–17 years old, respectively. Those authors performed linear multiple regression analyses for the total sample size, but they did not consider the differences between children and teenagers. Although age was considered an important independent factor in determining respiratory muscle strength, these studies applied the same final equations for the youngest and oldest volunteers. Considering the differences observed during childhood growth, we believe that it is necessary to determine distinct equations according to age in pediatric populations. In our opinion, it is not reasonable to group together individuals of such different stages of development.

The multiple regression employed in the present study identified age, BMI and the interaction between age and gender as variables that explain approximately 30% of the PImax and PEmax variations in Group 6–11. A similar R^2^ value was observed in Group 12–18, but age was not a variable that persisted in that model. We understand that factors other those included in our equation might influence respiratory muscle strength. Thorax size, diaphragm circumference and its contraction mechanics and hormones [24] are variables that may play an important role in predicting PImax and PEmax. These variables could be included in a model to increase the R^2^ value, but this was not done, as doing so would reduce the feasibility of applying the model successfully.

This final model includes interaction terms to show the effects of variables when another variable involved in the interaction has a value of zero. This is called a conditional effect and is very different from an unconditional effect, which is obtained when no product is included in the analysis. The interaction term between age and gender tested in this model was significant only in the Group 6–11. The interaction between height and weight, which was tested previously by Domenèch-Clar et al, [14] was not significant when tested in the present model. We also included a center dummy variable in the final model regression because we observed differences on the respiratory muscles strength between the centers. This happened because age was different between them. Even though we included dummy variable, the coefficients for the relevant variables (age, gender, BMI) only slightly change.

To our knowledge, Heinzman-Filho et al. [16] reported the best R^2^ value (0.58) for PImax and PEmax equations in a pediatric population (3–12 years old). The authors did not explore the reason for this higher R^2^ value. Another Brazilian equation, by Mendes et al [17] had an R^2^ value of 0.27, similar to our model. The R^2^ range for equations published of respiratory muscle strength in pediatric populations is 0.02–0.58. [12,14,16,17,22–25] Some factors that could justify this R^2^ range are the variability of techniques (volunteer position and lung volume) and the type of manometer.

In Brazil, two equations for respiratory muscle strength were developed [16,17]. Heinzmann-Filho [16] evaluated 171 volunteers and Mendes et al. [17] evaluated 182 volunteers. We compared the values of our sample with the predicted value from the Heinzman-Filho model (PImax: 90.4 ± 12.8cmH_2_O; PEmax: 102.7 ± 13.5cmH_2_O) and observed that our PImax (85.1 ± 21.9 cmH_2_O) and PEmax (85.1 ± 19.1 cmH_2_O) values were significant lower. We also compared the predicted values from the Mendes study (PImax: 87.6 ± 14.4 cmH_2_O; PEmax: 103.4 ± 17.1 cmH_2_O) to ours and observed that our sample demonstrated higher values for PImax (95.8 ± 19.0 cmH_2_O) and lower values for PEmax (92.1 ± 20.0 cmH_2_O). Some differences can be noted between the studies: Heinzman-Filho's study included volunteers between 4 and 12 years old and measured their respiratory muscles while sitting; and Mendes et al. evaluated respiratory muscle strength by a digital manometer (PImax and PEmax values were reached using manometer software) and they did not describe the reproducibility of the measurement. In addition, the sample sizes of the previous Brazilian studies were smaller than that of our study; both studies were conducted in a single center, whereas we conducted a multicenter study of 450 volunteers, which is more representative of the Brazilian pediatric population.

We did not evaluate other variables that could influence the respiratory muscle strength equation (hormones, nutrition, and body lean mass). To perform such evaluations, we would need specific equipment in all the centers, which was impractical. We did not perform lung function tests on the entire population as the required equipment was not available; however, all the volunteers’ parents completed a respiratory questionnaire, and volunteers with chronic or acute lung disease were excluded and there was no difference of respiratory muscle strength between the volunteers who did lung function and who did not.

In conclusion, we determined, in this multicenter study, a respiratory muscle prediction equation for children and teenagers. The variables that persisted in the equations were age, gender and BMI. The equations provided by this study can be used to interpret whether respiratory muscle strength is compromised when evaluating pediatric patients.

[5][11]
