# Correction: Reference Equation for Respiratory Pressures in Pediatric Population: A Multicenter Study

**DOI:** 10.1371/journal.pone.0146089

**Published:** 2015-12-23

**Authors:** Fernanda Cordoba Lanza, Mara Lisiane de Moraes Santos, Jessyca Pachi Rodrigues Selman, Jaksoel Cunha Silva, Natalia Marcolin, Jeniffer Santos, Cilmery M. G. Oliveira, Pedro Dal Lago, Simone Dal Corso

The following information is missing from the Funding section: This study was supported by the São Paulo Research Foundation (FAPESP) grant: 2015/15699-5.
